# Supplementary figures and images for: Reconstruction and Analysis of Thermodynamically Constrained Models Reveal Metabolic Responses of a Deep-Sea Bacterium to Temperature Perturbations
Source: mSystems. 2022 Aug 11;7(4):e00588-22. doi: 10.1128/msystems.00588-22 (PMC9426432; doi:10.1128/msystems.00588-22)

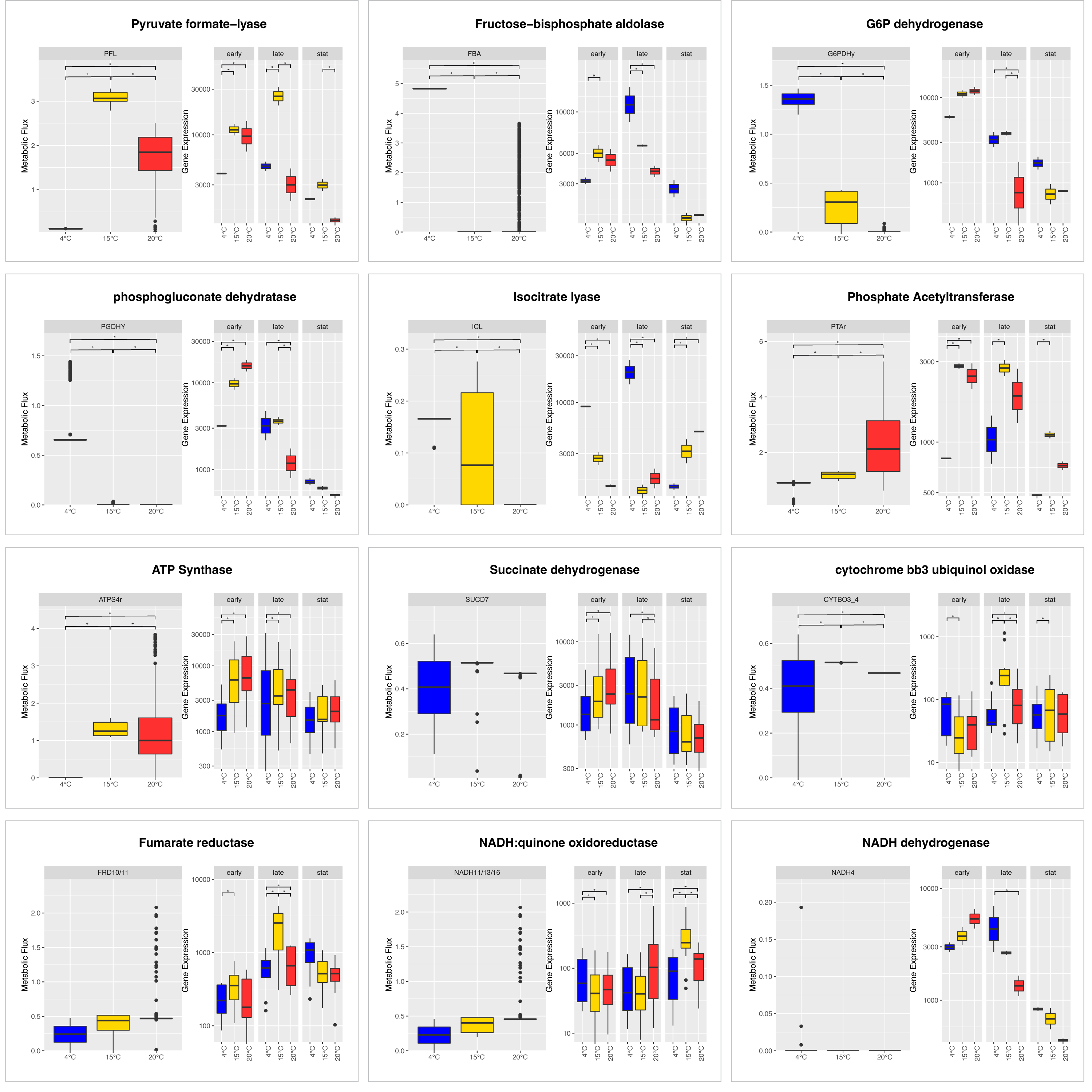

Supplement: FIG S1 [file msystems.00588-22-s0001.tif]

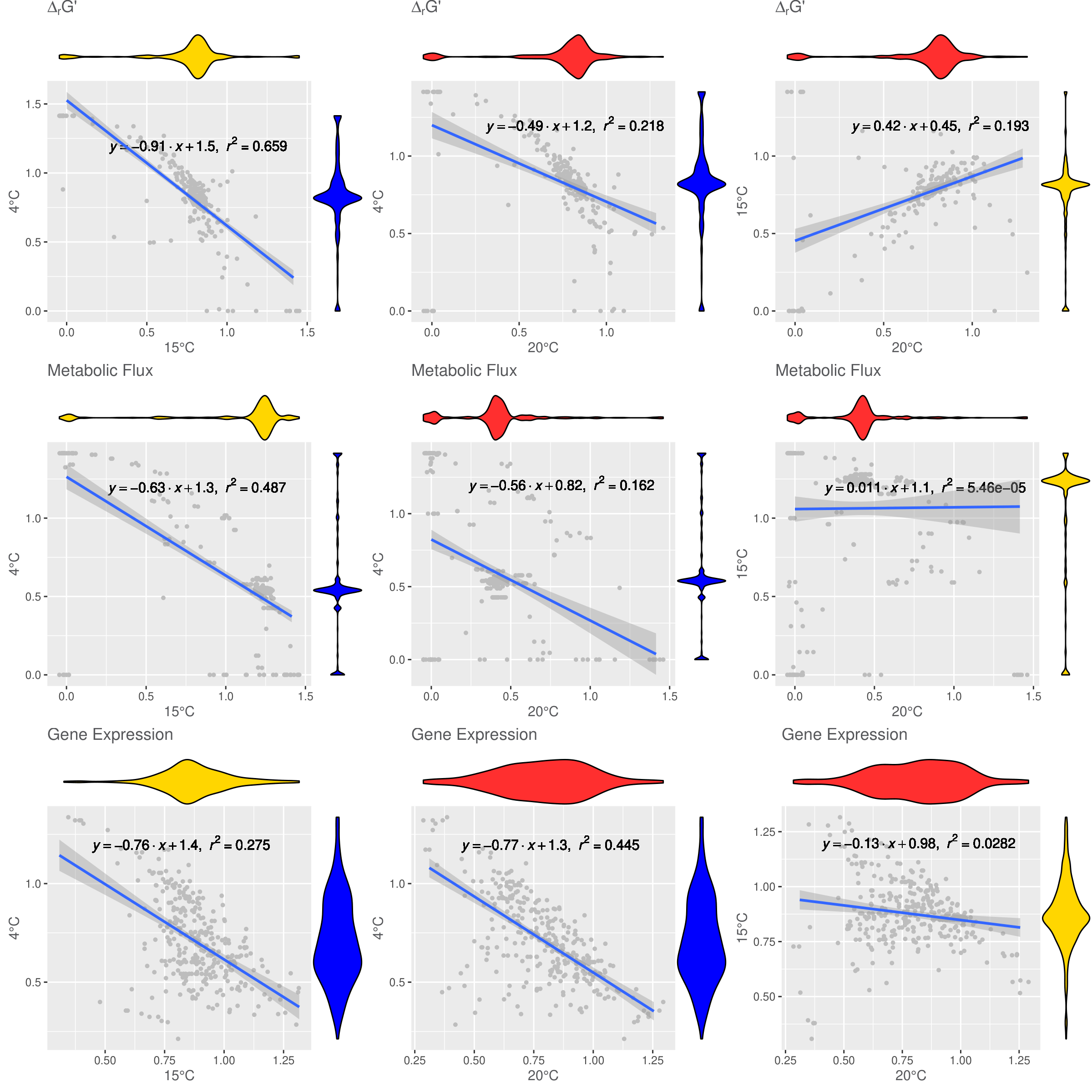

Supplement: FIG S2 [file msystems.00588-22-s0002.tif]
